# Supplementary material for: Different Dietary Ratios of Camelina Oil to Sandeel Oil Influence the Capacity to Synthesise and Deposit EPA and DHA in Zucker Fa/Fa Rats
Source: Nutrients. 2023 May 17;15(10):2344. doi: 10.3390/nu15102344 (PMC10224350; doi:10.3390/nu15102344)
Supplement: Supplementary file 1 [file nutrients-15-02344-s001.zip › nutrients-2375267-supplementary.pdf]

Supplementary

Table S1. Sequence, GenBank number and reference for primers used in qPCR analysis of rat liver.

Gene	Sequence (5'-3')	Genbank no	Reference
Fads1	GTTTGTGTGGGTGACGCAGAT<br>TTGAAGGCTGACTGGTGAACG	NM_053445	Zhou et al., 2008
Fads2	CGGGAGAAGATGCTACGGATG<br>GCCCTGAAGTCCTCGGTGAT	NM_031344	Zhou et al., 2008
Elovl5	CTCACCCTGCTGTCTCTCTA<br>ATCTGGTGGTTGTTCTTACG	NM_134382.1	Rodriguez-Cruz et al., 2011
Eif3a	CAGACTCCTGAGAGTGTTCTCTT<br>ACCCAGGGAGTCAGTAGCAA	NM_001047087	

Table S2. Biometric factors. Data are mean shown with standard deviation (BMI = body mass index, BW = body weight, Wat epi = epididymal visceral adipose tissue).

	Ctrl			CA			CA25+SA75			CA50+SA50			CA75+SA25			SA		
Weight, start (g)	434.3	±	15.8	425.0	±	15.7	421.0	±	11.1	425.3	±	18.5	420.8	±	24.1	427.7	±	6.4
Weight, final (g)	534.3	±	28.1	511.8	±	28.6	512.5	±	17.6	509.4	±	18.8	524.5	±	30.5	525.0	±	26.6
BMI	0.9	±	0.0	0.9	±	0.0	0.9	±	0.0	0.9	±	0.0	0.9	±	0.0	0.9		0.0
Liver/kg BW	55.1	±	4.5	54.0	±	5.4	54.3	±	2.9	62.1	±	11.9	55.2	±	4.3	58.6	±	7.9
Wat epi/kg BW	17.0	±	2.8	16.7	±	1.8	17.3	±	2.3	16.8	±	2.5	18.3	±	0.6	15.4	±	1.7

Table S3. Analyses of serum and plasma. Ctrl = control, CA = Camelina, SA = Sandeel. Data are means shown with standard deviation (n = 5-6). CRP = C-reactive protein, HDL = high-density lipoprotein, NEFA = non-esterified fatty acids, ALAT = alanine transaminase, ASAT = aspartate transaminase

	Ctrl	CA	CA75+SA25	CA50+SA50	CA25+SA75	SA
CRP (mg/ml)	0.33 ± 0.09	0.33 ± 0.05	0.32 ± 0.15	0.39 ± 0.11	0.37 ± 0.06	0.41 ± 0.08
Cholesterol (mmol/L)	8.3 ± 2.4	8.0 ± 2.7	7.5 ± 0.7	7.3 ± 1.1	8.3 ± 3.3	7.0 ± 0.7
HDL-cholesterol (mmol/L)	1.6 ± 1.9	1.7 ± 1.0	2.3 ± 1.5	2.3 ± 1.8	2.1 ± 1.4	2.4 ± 2.5
Triacylglycerol (mmol/L)	28.7 ± 17.3	28.0 ± 21.6	21.3 ± 9.6	18.3 ± 13.2	21.0 ± 15.8	17.7 ± 12.3
NEFA (mmol/L)	0.55 ± 0.24	0.58 ± 0.25	0.56 ± 0.10	0.48 ± 0.15	0.40 ± 0.24	0.36 ± 0.03
Bile acids (umol/L)	43 ± 22	32 ± 9	51 ± 29	40 ± 20	38 ± 19	35 ± 16
ALAT (U/L)	91 ± 24	82 ± 32	91 ± 16	96 ± 28	81 ± 19	89 ± 30
ASAT (U/L)	93 ± 49	84 ± 28	85 ± 20	104 ± 25	90 ± 19	103 ± 51

Table S4: Fatty acid composition (% of total fatty acids) in blood cells. Data are means (n=6) shown with standard deviation. Different letters indicate significant differences ($p<0.05$) evaluated using one-way ANOVA followed by Tukey's HSD. Ctrl = Control, CA = Camelina oil, SA = Sandeel oil.

	Ctrl	CA	CA75+SA25	CA50+SA50	CA25+SA75	SA
14:0	0.63 ± 0.2	0.56 ± 0.1	0.70 ± 0.2	0.61 ± 0.3	0.82 ± 0.2	0.76 ± 0.3
16:0	25.91 ± 2.3	25.90 ± 2.1	25.63 ± 0.9	25.52 ± 1.4	26.00 ± 0.9	26.44 ± 1.4
18:0	11.73 ± 2.4	12.02 ± 2.4	11.97 ± 1.4	12.24 ± 2.6	10.84 ± 1.8	11.46 ± 1.9
24:0	0.50 ± 0.2	0.47 ± 0.1	0.49 ± 0.1	0.59 ± 0.2	0.46 ± 0.1	0.53 ± 0.2
**ΣSFA**	39.26 ± 1.2	39.43 ± 1.5	39.30 ± 0.8	39.54 ± 1.4	38.63 ± 1.8	39.72 ± 1.4
16:1n-7	3.01 ± 1.0	2.61 ± 0.6	2.68 ± 0.6	2.56 ± 1.5	3.36 ± 0.5	3.21 ± 1.3
18:1n-9	16.75 ± 4.4	16.16 ± 5.0	15.07 ± 1.9	13.34 ± 5.0	15.82 ± 3.2	14.28 ± 3.6
18:1n-7	2.93 ± 0.3 a	2.48 ± 0.3 ab	2.35 ± 0.1 b	2.42 ± 0.3 b	2.47 ± 0.1 ab	2.49 ± 0.2 ab
20:1n-9	0.26 ± 0.1 b	0.91 ± 0.3 a	0.90 ± 0.2 a	0.78 ± 0.3 a	0.85 ± 0.1 a	0.69 ± 0.2 a
24:1n-9	0.30 ± 0.1 b	0.51 ± 0.1 ab	0.52 ± 0.1 ab	0.67 ± 0.3 a	0.49 ± 0.1 ab	0.58 ± 0.2 ab
**ΣMUFA**	23.70 ± 5.6	23.09 ± 6.0	21.94 ± 2.7	20.14 ± 6.8	23.40 ± 3.8	21.62 ± 5.1
18:2n-6	12.30 ± 2.2 a	9.55 ± 0.9 b	9.56 ± 0.9 b	9.09 ± 1.5 b	9.64 ± 0.2 b	8.58 ± 0.9 b
20:3n-6	0.73 ± 0.1 c	0.78 ± 0.1 bc	0.83 ± 0.1 abc	0.78 ± 0.1 bc	1.00 ± 0.1 a	0.97 ± 0.0 ab
20:4n-6	15.53 ± 5.6	15.13 ± 4.8	15.28 ± 3.4	16.50 ± 6.1	12.27 ± 2.1	13.83 ± 5.0
22:4n-6	1.03 ± 0.2 a	0.63 ± 0.1 b	0.55 ± 0.1 b	0.57 ± 0.2 b	0.44 ± 0.1 b	0.47 ± 0.1 b
22:5n-6	0.75 ± 0.1 a	0.31 ± 0.1 b	0.29 ± 0.0 b	0.28 ± 0.1 b	0.24 ± 0.0 b	0.27 ± 0.1 b
**Σn-6 PUFA**	30.75 ± 3.9	26.86 ± 4.3	26.93 ± 2.5	27.53 ± 4.8	23.92 ± 2.2	24.37 ± 4.3
18:3n-3	0.70 ± 0.2 c	2.21 ± 0.7 a	2.01 ± 0.7 ab	1.38 ± 0.8 abc	1.13 ± 0.3 bc	0.44 ± 0.2 c
20:5n-3	0.17 ± 0.0 d	0.71 ± 0.2 cd	1.17 ± 0.2 bc	1.60 ± 0.2 b	2.51 ± 0.5 a	2.70 ± 0.5 a
22:5n-3	0.84 ± 0.2 b	1.75 ± 0.4 a	1.80 ± 0.1 a	1.94 ± 0.2 a	2.10 ± 0.2 a	2.17 ± 0.2 a
22:6n-3	1.99 ± 0.4 c	3.05 ± 0.7 b	3.85 ± 0.3 b	4.12 ± 0.5 b	5.33 ± 0.9 a	5.53 ± 0.4 a
**Σn-3 PUFA**	3.70 ± 0.5 d	7.86 ± 1.1 c	8.99 ± 1.0 c	9.11 ± 1.1 bc	11.28 ± 1.0 a	11.00 ± 1.2 ab

Table S5. Fatty acid composition (% of total fatty acids) and fat content (%) in liver. Data are means shown with standard deviation (n=6). Different letters indicate significant differences (p<0.05) evaluated using one-way ANOVA followed by Tukey's HSD. CA = Camelina, SA = Sandeel.

	Ctrl				CA				CA75+SA25				CA50+SA50				CA25+SA75				SA			
**Fat %**	13.5	±	6.8		11.2	±	4.0		11.8	±	4.6		15.2	±	6.1		12.2	±	1.1		14.3	±	6.1	
**14:0**	1.2	±	0.2		1.1	±	0.1		1.2	±	0.2		1.1	±	0.2		1.3	±	0.2		1.3	±	0.1	
**16:0**	31.7	±	1.4		31.4	±	1.0		31.9	±	1.4		31.5	±	1.8		30.2	±	4.6		31.4	±	1.4	
**18:0**	7.7	±	2.3		8.4	±	1.5		8.4	±	2.3		6.4	±	2.3		6.5	±	1.4		6.3	±	1.2	
**ΣSFA**	41.0	±	1.4		41.3	±	0.9		41.9	±	1.2		39.4	±	1.8		38.4	±	6.0		39.5	±	1.0	
**14:1 n-5**	0.3	±	0.1		0.2	±	0.1		0.3	±	0.1		0.3	±	0.1		0.3	±	0.0		0.3	±	0.1	
**16:1 n-9**	0.6	±	0.1		0.5	±	0.0		0.4	±	0.3		0.6	±	0.1		0.5	±	0.1		0.5	±	0.1	
**16:1 n-7**	6.1	±	1.5		5.2	±	0.5		5.6	±	1.2		5.9	±	0.9		6.2	±	1.2		6.6	±	0.7	
**18:1 n-9**	25.2	±	4.4		24.4	±	4.4		24.6	±	4.7		27.6	±	6.4		23.9	±	4.0		25.6	±	5.6	
**18:1 n-7**	3.4	±	0.5	a	2.8	±	0.3	ab	2.7	±	0.3	b	2.9	±	0.2	ab	2.6	±	0.5	b	2.9	±	0.1	ab
**20:1 n-9**	0.1	±	0.0	b	0.4	±	0.0	a	0.3	±	0.1	a	0.3	±	0.1	a	0.3	±	0.1	a	0.4	±	0.1	a
**ΣMUFA**	36.0	±	6.3		33.8	±	4.6		34.1	±	6.1		37.9	±	6.9		34.3	±	5.5		37.0	±	5.8	
**18:2 n-6**	8.8	±	0.8	a	7.8	±	0.5	ab	7.2	±	0.4	ab	7.3	±	0.9	ab	6.3	±	1.4	b	7.3	±	1.2	ab
**20:3 n-6**	0.5	±	0.2		0.6	±	0.1		0.7	±	0.2		0.5	±	0.3		0.6	±	0.2		0.7	±	0.3	
**20:4 n-6**	7.0	±	2.6		6.9	±	1.9		6.4	±	2.3		4.6	±	2.3		4.1	±	1.5		3.9	±	1.1	
**22:4 n-6**	0.4	±	0.2	a	0.2	±	0.0	b	0.1	±	0.0	bc	0.1	±	0.1	bc	0.0	±	0.0	c	0.1	±	0.1	bc
**Σn-6 PUFA**	17.3	±	3.5	a	15.9	±	2.4	ab	14.8	±	2.9	ab	12.8	±	3.2	ab	11.4	±	3.1	b	12.3	±	2.5	ab
**18:3 n-3**	0.4	±	0.1	e	1.8	±	0.2	a	1.4	±	0.2	ab	1.1	±	0.3	bc	0.8	±	0.2	cd	0.5	±	0.1	de
**20:5 n-3**	0.1	±	0.0	c	0.4	±	0.1	bc	0.6	±	0.2	bc	0.7	±	0.3	b	0.9	±	0.2	b	1.5	±	0.6	a
**22:5 n-3**	0.3	±	0.1	c	0.9	±	0.2	bc	0.9	±	0.2	b	0.9	±	0.3	bc	0.9	±	0.3	b	1.5	±	0.5	a
**22:6 n-3**	2.2	±	0.9	b	3.4	±	0.9	ab	3.9	±	1.2	ab	3.5	±	1.5	ab	4.1	±	1.4	ab	5.5	±	1.8	a
**Σn-3 PUFA**	3.1	±	1.1	b	6.7	±	1.3	ab	6.9	±	1.7	a	6.3	±	2.4	ab	6.8	±	1.9	a	9.1	±	3.1	a
**EPA+DHA**	2.3	±	0.9	b	3.9	±	1.1	b	4.5	±	1.3	ab	4.2	±	1.8	ab	5.0	±	1.5	ab	6.9	±	2.4	a
**n-6/n-3**	5.9	±	1.3	a	2.4	±	0.2	b	2.2	±	0.2	b	2.1	±	0.4	b	1.7	±	0.1	b	1.5	±	0.5	b

Table S6. Fatty acid composition (% of total fatty acids) and fat content (%) in heart. Data are means shown with standard deviation (n=6). Different letters indicate significant differences ($p<0.05$) evaluated using one-way ANOVA followed by Tukey's HSD. CA = Camelina, SA = Sandeel.

	Ctrl	CA	CA75+SA25	CA50+SA50	CA25+SA75	SA
**Fat %**	4.2 ± 0.2	3.9 ± 0.8	4.6 ± 0.9	4.1 ± 1.0	4.8 ± 1.2	4.02 ± 0.4
**14:0**	0.3 ± 0.1	0.3 ± 0.1	0.6 ± 0.2	0.4 ± 0.4	0.6 ± 0.3	0.5 ± 0.1
**16:0**	15.6 ± 0.8	15.8 ± 1.5	17.9 ± 2.3	16.5 ± 2.7	17.8 ± 2.0	16.8 ± 0.5
**18:0**	17.4 ± 0.9	16.9 ± 1.3	15.2 ± 2.2	15.7 ± 2.3	14.7 ± 2.0	15.8 ± 0.8
**ΣSFA**	33.5 ± 0.2	33.2 ± 0.7	33.9 ± 0.5	32.9 ± 1.0	33.3 ± 0.4	33.4 ± 0.7
**16:1 n-7**	1.3 ± 0.3	1.4 ± 0.7	2.5 ± 1.1	1.9 ± 1.3	2.5 ± 1.2	1.8 ± 0.4
**18:1 n-9**	8.7 ± 1.3	9.2 ± 2.2	11.4 ± 3.3	9.8 ± 3.4	10.9 ± 3.4	9.1 ± 1.4
**18:1 n-7**	4.0 ± 0.2 a	3.7 ± 0.2 b	3.7 ± 0.1 b	3.6 ± 0.1 b	3.5 ± 0.1 b	3.7 ± 0.1 b
**20:1 n-9**	0.1 ± 0.0 c	0.6 ± 0.0 a	0.6 ± 0.0 a	0.6 ± 0.1 ab	0.5 ± 0.1 ab	0.5 ± 0.1 b
**22:1 n-11**	0.0 ± 0.0 c	0.0 ± 0.0 c	0.0 ± 0.0 c	0.2 ± 0.2 bc	0.5 ± 0.1 ab	0.7 ± 0.4 a
**ΣMUFA**	14.2 ± 1.5	15.0 ± 2.8	18.5 ± 4.7	16.4 ± 5.1	18.3 ± 4.9	16.1 ± 1.8
**18:2 n-6**	12.9 ± 0.8	12.3 ± 0.5	12.6 ± 0.5	12.9 ± 1.0	12.6 ± 1.1	12.0 ± 0.3
**20:2 n-6**	0.2 ± 0.1 b	0.4 ± 0.1 a	0.3 ± 0.0 ab	0.3 ± 0.0 ab	0.2 ± 0.0 b	0.0 ± 0.1 c
**20:3 n-6**	0.7 ± 0.1	0.8 ± 0.1	0.7 ± 0.1	0.7 ± 0.2	0.7 ± 0.1	0.8 ± 0.1
**20:4 n-6**	18.7 ± 1.0 a	16.3 ± 1.7 ab	13.8 ± 2.3 bc	14.3 ± 2.4 bc	12.1 ± 1.8 c	13.2 ± 0.7 c
**22:4 n-6**	1.8 ± 0.2 a	0.7 ± 0.1 b	0.5 ± 0.1 c	0.4 ± 0.1 cd	0.3 ± 0.0 cd	0.2 ± 0.1 d
**22:5 n-6**	2.7 ± 0.4 a	0.7 ± 0.1 b	0.4 ± 0.1 bc	0.4 ± 0.0 bc	0.3 ± 0.0 c	0.2 ± 0.1 c
**Σn-6 PUFA**	37.1 ± 0.8 a	31.1 ± 1.9 b	28.2 ± 2.2 bc	28.9 ± 2.7 bc	26.2 ± 2.0 c	26.5 ± 0.8 c
**18:3 n-3**	0.4 ± 0.1 de	1.2 ± 0.2 ab	1.4 ± 0.3 a	0.9 ± 0.3 bc	0.7 ± 0.2 cd	0.4 ± 0.1 e
**20:5 n-3**	0.1 ± 0.0 d	0.2 ± 0.0 cd	0.4 ± 0.1 bc	0.5 ± 0.1 b	0.8 ± 0.1 a	0.9 ± 0.2 a
**22:5 n-3**	2.7 ± 0.2 c	4.8 ± 0.4 a	3.6 ± 0.5 b	3.7 ± 0.7 b	3.5 ± 0.6 bc	3.6 ± 0.4 b
**22:6 n-3**	7.9 ± 1.1 c	10.4 ± 1.0 bc	10.6 ± 2.0 bc	12.6 ± 2.4 ab	13.5 ± 2.5 ab	15.2 ± 1.4 a
**Σn-3 PUFA**	11.1 ± 0.9 c	16.7 ± 1.0 ab	15.9 ± 2.2 b	17.7 ± 2.8 ab	18.5 ± 2.8 ab	20.1 ± 1.9 a
**EPA+DHA**	8.0 ± 1.0 c	10.6 ± 1.0 bc	11.0 ± 2.1 bc	13.1 ± 2.4 ab	14.3 ± 2.5 a	16.2 ± 1.6 a
**n-6/n-3**	3.4 ± 0.2 a	1.9 ± 0.1 b	1.8 ± 0.2 b	1.7 ± 0.2 bc	1.4 ± 0.1 c	1.3 ± 0.2 c
